# The Failing Heart

**Published:** 1907-12-28

**Authors:** S. Russell Wells

**Affiliations:** Senior Assistant Physician to the Seamen's Hospital Greenwich; Lecturer on the Practice of Medicine, London School of Clinical Medicine; Assistant Physician to the National Hospital for Diseases of the Heart.


### Hospital Clinics.

### THE FAILING HEART.

By S. RUSSELL WELLS, M.D., B.Sc., M.R.C.P., Senior Assistant Physician to the Seamen's Hospital Greenwich; Lecturer on the Practice of Medicine, London School of Clinical Medicine; Assistant Physician to the National Hospital for Diseases of the Heart.

A lecture delivered at the Polyclinic and Medical Graduates College.

Gentlemen,—I propose this afternoon to discuss certain cases and conditions perhaps best grouped under the title of the failing heart. I do not intend, however, to deal with the whole subject of heart failure, but only with that aspect of "failing compensation" such as no doubt you all frequently see in your own practices.

I think I can best begin by giving you a few typical histories. Three years ago I saw a girl aged twenty, who had never had rheumatic fever, and, as far as she knew, had been in perfect health until two months before, when, while racing some of her companions on a bicycle up a steep hill she was seized with severe pain in the chest, intense dyspnœa and palpitation, and faintness; she was with great difficulty taken home by her companions. She spent three weeks in bed, during which time the dyspnœa and palpitation slowly subsided, and she learnt from her doctor that she had a systolic murmur. When I saw her at the Hospital for Diseases of the Heart two months after the bicycle ride in question, she had an apical systolic murmur, no marked dilatation of the heart, a rather rapid pulse, slight dyspnœa on exertion, and occasional attacks of palpitation. She was given iron, advised to rest as much as possible, and to avoid various things likely to put a strain upon the heart.

The murmur gradually changed in character, becoming a typical presystolic murmur, a thrill became palpable at the apex, and all the signs of mitral stenosis appeared. Compensation, however, was established, and for nearly two years, though she could not take without discomfort the violent exercise she had previously done, her heart in no way seriously inconvenienced her. Her occupation was that of a pay-desk clerk in a shop. She now got into a very hard situation, where the work was excessive and trying, and the hours inordinately long. She began to complain of always feeling tired, she became somewhat anæmic, and got attacks of palpitation and dyspnœa on slight provocation; slight œdema of the legs appeared, and after a time the heart began to dilate, the loud presystolic murmur gradually giving place to a soft systolic one. However, rest in bed with digitalis and strychnine restored her to her previous condition, the heart gained in force, the presystolic murmur and thrill reappeared, and having got an easier situation she has been for the last ten months an ordinary case of compensated mitral stenosis.

What is the interpretation of this case? It is possible that before the bicycle ride there was a lesion of the mitral valve of which the patient was ignorant; it is also possible that there was an actual rupture of the valve at the time of the ride, with subsequent healing and contraction, leading to narrowing of the orifice; however, this question need not detain us now. The important point for our present purpose is that, though she had undoubted stenosis, for two years it gave her comparatively little inconvenience until she was overworked, and that after the adverse circumstances were removed she reacquired a condition of what she considers health. Now, it is absurd to suppose that the amount of stenosis is materially less at the present time than it was at the time of her breakdown, or that it suddenly became greater just before this last attack; therefore we must look to something other than the valvular lesion and its immediate effects to account for her failing heart. Of course, what was at fault was the cardiac muscle.

Let us now take another case. I recently saw at the Seamen's Hospital a sailor aged thirty-seven, who complained of palpitation, pain in the region of the heart, shortness of breath, and swelling of the legs. Examination showed that the cardiac dullness extended from an inch to the right of the sternum to half an inch outside the left nipple line, a presystolic murmur and a thrill were present, the liver dullness was increased, and there were coarse rales in the lungs. He had rheumatic fever when aged about fourteen, and was then told that his heart was affected, but he got over it completely, and never had a day's illness until his present symptoms came on between three weeks and a month previously. He had just come off a sailing ship where they had been short-handed in very bad weather, and were, in his own words, "very lucky to get through." He thought "the constant wettings, bad food, and heavy hauling were what had knocked him over." Here probably the stenosis had its origin twenty-three years before, but, being compensated, had give rise to no trouble until this time of stress and strain occurred.

If you go to the museum of any of our large hospitals and look through the preparations, you are sure to find several showing extreme stenosis of the mitral orifice, and a careful examination will, I think, convince you that many of them have been of very long standing. In comparatively few will you see any signs of recent change in the valve; in fact, it is remarkable what an extreme degree of deformity is apparently consistent with fair health. If you read the clinical histories of the specimens in question, you are bound to be struck by the apparent want of connection between the lesion and the proximate cause of the final cardiac failure. I have used two cases of mitral stenosis as illustrations, but the same applies more or less to other valvular lesions, that is, it is comparatively rare for them to be directly the cause of death. I have on the table two specimens of aortic stenosis, and if you look at them you will see that the stenosis must have existed practically unchanged for a very long time before the patient died. There is one of my out-patients at the Hospital for Diseases of the Heart whom I first saw five years ago with a double aortic and a double mitral murmur; his heart is so enormous that it has deformed his chest, the left side being notably larger than the right; but in spite of these very grave valvular lesions he is able to pursue his trade of a cardboard box maker, which entails his constantly cutting through piles of cardboard three inches thick by means of a guillotine worked by his foot. I must admit when I first saw him I estimated his expectation of life at a very few months, but though he has had one or two slight breakdowns he is working every day of his life and supporting a wife and family, and, as far as I can judge, is no worse than he was five years ago.

Now let us discuss what actually occurs when the heart beats. In order to be clear we will suppose that the quantity of blood which the left auricle discharges into the left ventricle at each systole is four ounces; then, when the ventricle begins to contract, the mitral valve is closed and this blood is forced through the aortic orifice into the aorta, while the same quantity of blood leaves the venous end of the systemic circulation and enters the pulmonary system *via* the right side of the heart. Hence each of the cardiac cavities must discharge exactly the same quantity, for if the left ventricle regularly discharged but one drop more per beat into the aorta than the right auricle discharged into the right ventricle (the tricuspid valve being efficient), in a short time all the blood would be in the systemic vessels.

Let us now suppose that a lesion occurs to one of the valves—say, for the sake of example, one of the flaps of the mitral valve—and that the resulting regurgitation is such that at the next beat of the heart after this happens the left ventricle in contracting forces one ounce of blood back into the left auricle and only three ounces on into the aorta; the result is that the systemic system gets less blood than before, while the pulmonary side has an ounce too much. Presently the great veins will discharge less blood into the right auricle, and so the right ventricle will discharge less than four ounces of blood into the pulmonary arteries, and consequently the pressure in the pulmonary circulation will rise rather less than might at first sight be supposed. In consequence of this rise the pulmonary veins will during the auricular diastole discharge more than the four ounces they previously did into the left auricle, and the auricle will discharge more than four ounces into the left ventricle; consequently a time will be reached when, on our supposition, five and a third ounces of blood are discharged at each beat into the left ventricle, and if as before a quarter of the total quantity is regurgitated four ounces will again be forced into the aorta. Hence it is possible, without any alteration in the heart's rate, for mitral regurgitation to be compensated by rise of pressure in the pulmonary system. The result of this increased pressure is that the left auricle tends to dilate in order to accommodate the increased quantity of blood, and afterwards to hypertrophy. But there is another important effect. The right ventricle is called upon to do more work in forcing its quantum of blood into the pulmonary arteries, and it too hypertrophies.

It has been assumed in what I have said that the rate of heartbeat remains the same, but it is easy to see that so far as the general circulation is concerned, with diminished delivery into the aorta the same flow might be maintained through the systemic vessels by an increased rate of heartbeats without an increased delivery of blood into the left ventricle, but here again the ultimate effect is increased work on the cardiac muscle. Similar lines of argument will show that the ultimate effect of any valvular lesion is to throw increased work on the muscle of the heart, if the circulation is to be efficiently maintained. We may look upon the work the heart has to do as being measured by the amount of resistance it has to overcome in order to maintain the flow at the requisite rate. So far we have considered valvular lesions alone, but a little thought will show that a similar strain on the heart muscle may be brought about in other ways. Let me give you the history of a case which represents a considerable class. I saw at the Seamen's Hospital some months ago a man of sixty-two years of age, who complained of shortness of breath, and some œdema of the legs, but not excessive, nor of that white, puffy character that one usually associates with renal disease. He had a slight cough, and complained of palpitation; the pulse was very irregular, the urine contained a fair amount of albumen, the appearance of his face suggested that he had granular kidney, and he had for years passed a large quantity of pale urine. On examination I found that there were a few râles over the back of the lungs, a small quantity of fluid in both pleural cavities, the liver was enlarged, and there was possibly some ascites. His heart was greatly enlarged, and there was a faint apical systolic murmur, while the sounds at the base were faint and had a tick-tack character. I diagnosed the case as one of renal disease and failing heart similar to that of the latter stages of mitral regurgitation. I took the patient into the Hospital, purged him, gave him strophanthus and strychnine, treating him in much the same way one would a failing heart from mitral disease. The diagnosis and treatment were justified by the result; the man steadily improved, his œdema disappeared, and the heart's action became more regular.

What is the explanation of this case? As the result of his granular kidney, which was no doubt the primary affection, the tension in the systemic vessels rose; we need not stop here to enquire the exact means by which this rise in tension is brought about, but as a result the left ventricle had more work to do in forcing the blood into the aorta. As the tension slowly rose, so the ventricle hypertrophied to meet it, while the muscle could respond to the call upon it; all went well as far as the heart was concerned, but a time came when the ventricle was no longer capable of standing the pressure it was subjected to, and must perforce dilate. The mitral valve then became relatively incompetent, and the case, though originally one of renal disease, presented the classical signs of a failing heart due to mitral reflux.

This raises an interesting point. Can the normal work of the heart ever be too much for the muscle? In other words, can the symptoms we have been discussing be brought about by changes in the muscle itself? No doubt, as a rule, we associate muscular changes in the heart with more sudden symptoms than obtain in the cases we have been discussing; in the degeneration one sees in diphtheria, for example, sudden death occurs, and no doubt the heart failure in other acute infective diseases is frequently due to a poisoning of the heart muscle. We are also apt to associate fatty degeneration of the heart with sudden death, but there are undoubtedly cases where a slowly failing heart results from primary degeneration of the muscle. I will cite such a case from my own experience. Some four years ago I saw a gentleman who had an attack of acute palpitation. He was then about forty-seven years of age. I found him cyanosed and dyspnœic without œdema, but with an extremely irregular and intermittent pulse. On percussion the heart's apex was found to be an inch outside the nipple line, but the beat was so feeble and diffuse that the apex could not be accurately localised by palpation; most careful auscultation failed to reveal any murmur; there was no history of any illness likely to cause a valvular lesion, nor could I find anything definitely pointing to one. A careful examination failed to reveal any symptoms or signs of renal disease. The patient was ordered complete rest, and by careful treatment recovered from his acute symptoms, but the pulse remained irregular and the heart continued large. He went through a Nauheim course later, and completely recovered so far as his own feelings went. His pulse became regular, and he was capable of exertion without dyspnœa or fatigue; indeed, he not infrequently indulged in long walks without ill effects. There were no signs of arterial or renal trouble, but the heart remained somewhat large. Three years after his attack he had a slight febrile ailment, and again had attacks of palpitation, dyspnœa, and cyanosis; the pulse became extremely irregular, and, as he was a nervous and excitable individual, it was impossible to keep him at rest. After a time œdema of the legs appeared, and later ascites, but still there was no murmur of any sort to be heard in the heart. The condition got steadily worse for two months, until he presented all the symptoms of a mitral case with broken-down compensation but no systolic murmur was audible. He now consented to remain in bed, was energetically purged with compound jalap powder, and given two-drachm doses of infusion of digitalis every four hours, and strychnine. The result was most satisfactory. The cardiac dulness after a month of such treatment shrank to just within the nipple line, œdema and ascites entirely disappeared, and the pulse, which had been 150 to the minute, and at times so irregular and intermittent as to be absolutely uncountable, sank to eighty and became regular. He now insisted on getting out of bed and driving about the country, and in spite of advice his intense excitability and nervousness prevented him remaining quiet. After six weeks he was again as bad as ever, and in spite of all treatment died some four weeks later. I had the opportunity of inspecting the heart and kidneys twenty-four hours after death. The kidneys showed the usual appearances of a chronic congestion, but nothing more. The heart was very greatly hypertrophied and dilated, all the valves were absolutely healthy and competent, but the muscle was in a condition that might be compared to that of wet brown paper, for the finger could be readily passed through it anywhere. Microscopic examination showed that the muscle fibres were full of droplets of fat; there were granules of brown pigment between them and an increase of fibrous tissue; the striation of the muscle was for the most part lost, and the protoplasm was broken up into short segments.

Let us now see what we can learn from the cases I have quoted. The first and most obvious lesson is that grave defects of the valves may exist for many years without seriously interfering with the comfort or occupation of the patient, but these defects entail increased work upon the muscle of the heart, and it is not until the muscle begins to give way that we get that group of symptoms which we associate with the failing heart. I have also tried to show that the same group of symptoms may be caused by the heart muscle becoming incapable of doing the extra amount of work required of it as the result of increased resistance outside the heart, as in renal disease. Further, we find that the same set of symptoms may be produced by the normal work of the heart being too great for a degenerated muscle.

Where we are dealing with a case of valvular lesion a comparatively slightly degeneration of the muscle is sufficient to produce these symptoms. We should therefore expect to find, where a patient has died from a failing heart, that, other things being equal, the amount of muscular degeneration would be in inverse proportion to the gravity of the valvular defect. In the case of death as the result of renal disease we should expect more change in the muscle, while if the symptoms had been caused by degeneration of the muscle alone, we should expect to find very marked changes. On the whole, microscopic examination bears out these anticipations. Since we have been led to the conclusion that the condition of the cardiac muscle is what in the majority of these cases determines the question of life or death, it is extremely important to investigate the various conditions likely to bring about muscular degeneration. When the heart muscle has to contend with greatly impaired valves and is only just equal to maintaining the circulation, a sudden increase of the peripheral pressure may overthrow the balance and cause the tissue destruction to be in excess of the power of repair, or, if you prefer the phrase, the catabolic processes to be in excess of the anabolic; and since the cardiac muscle is already taxed almost to its uttermost, repair will be extremely slow. We find that severe and unaccustomed muscular exercise, such as bicycling up hill, increased work, straining at stool, or the like, by raising the general blood pressure, may be the immediate cause of a breakdown; therefore we should caution our cardiopaths against such things. I do not, of course, mean that suitable and graduated muscular exercise is to be avoided; indeed, this may promote the nutrition of the cardiac muscle; but that severe and intermittent strain is to be guarded against.

Over-feeding is another undoubted cause of muscular degeneration, and is particularly injurious in heart cases, not only because waste products tend to produce fatty degeneration of the muscle, but also because over-eating, by increasing the quantity of blood the heart has to deal with, throws increased work upon its muscle. Time does not permit my entering in detail into the question of diet in these cases, but, speaking generally, I may remark that the diet should be light, easy of digestion, and not bulky; it should not consist too largely of articles containing the so-called extractives.

Allied to the question of food is that of alcohol. Here we are dealing with a direct muscle poison, having an effect in producing fatty degeneration comparable to that of phosphorus. Cardiac patients are apt to be depressed, and somewhat dyspeptic, and they have, in consequence, a great temptation to take stimulants rather freely to relieve these symptoms. It cannot be too emphatically impressed upon them that the relief so gained is only temporary, and the injury permanent. I do not mean that we should invariably insist that those who have all their life been in the habit of taking a small and moderate amount of stimulant must necessarily become teetotalers, but only to emphasise the deleterious effect of alcoholic excess on the cardiac muscle, and to point out that an amount of injury to the heart muscle by alcohol which in more normal individuals would pass unnoticed may in sufferers from valvular disease prove fatal.

Yet another cause of muscular degeneration is anæmia, which alone may lead to a certain amount of fatty degeneration. Since valvular defects are them selves liable, by interfering with the pulmonary circulation, to impede the proper oxygenation of the blood, a vicious circle is easily set up; hence the value of iron in chronic heart cases. A patient with enfeebled action of the heart must be guarded against anæmia, and warned to avoid close rooms, unsuitable diet, and other unhygienic conditions which tend to diminish the hæmoglobin in the blood. Constipation, besides the mechanical effect it produces of straining is probably harmful by causing the elaboration of various toxins inimical to the muscular tissue.

However, probably the most active muscular poisons with which we have to deal are those of certain infective diseases. I have under the microscope here a section of heart muscle from the case of a patient who died from sudden cardiac failure after diphtheria, and if you look at it you will see numerous patches of fatty degeneration. It is well known that patients who die from febrile diseases often show the condition known as cloudy swelling of the heart muscle, and there is ample clinical evidence that the onset of the symptoms of failing heart is frequently preceded by some slight febrile disturbance. I therefore urge you to treat any slight infection in a cardiopath as a grave illness, and to be on the look-out for the earliest symptoms of heart failure so as to deal with them at once. In giving a prognosis in a heart case, determine what the condition of the valves may be, but attach infinitely more importance to the size of the heart and the condition of its muscle as far as you can gauge it, and take into consideration those factors in the patient's surroundings and habits which make for healthy muscle or the reverse. Remember that very grave valvular lesions are not incompatible with many years of life, and that it is the muscle which is the efficient, active, and living thing about the heart.

